# Lignin-Based Mesoporous Hollow Carbon*@*MnO_2_ Nanosphere Composite as an Anodic Material for Lithium-Ion Batteries

**DOI:** 10.3390/ma16237283

**Published:** 2023-11-23

**Authors:** Shun Li, Jianguo Huang, Guijin He

**Affiliations:** 1 College of Chemistry and Materials Engineering, Zhejiang A&F University, Hangzhou 311300, China; 2 Department of Chemistry, Zhejiang University, Hangzhou 310058, China; jghuang@zju.edu.cn

**Keywords:** hollow carbon nanospheres, lignin, MnO_2_ nanoparticles, nanoarchitecture, lithium-ion batteries

## Abstract

The lignin-based mesoporous hollow carbon*@*MnO_2_ nanosphere composites (L-C-NSs*@*MnO_2_) were fabricated by using lignosulfonate as the carbon source. The nanostructured MnO_2_ particles with a diameter of 10~20 nm were uniformly coated onto the surfaces of the hollow carbon nanospheres. The obtained L-C-NSs*@*MnO_2_ nanosphere composite showed a prolonged cycling lifespan and excellent rate performance when utilized as an anode for LIBs. The L-C-NSs*@*MnO_2_ nanocomposite (24.6 wt% of MnO_2_) showed a specific discharge capacity of 478 mAh g^−1^ after 500 discharge/charge cycles, and the capacity contribution of MnO_2_ in the L-C-NSs*@*MnO_2_ nanocomposite was estimated ca. 1268.8 mAh g^−1^, corresponding to 103.2% of the theoretical capacity of MnO_2_ (1230 mAh g^−1^). Moreover, the capacity degradation rate was ca. 0.026% per cycle after long-term and high-rate Li^+^ insertion/extraction processes. The three-dimensional lignin-based carbon nanospheres played a crucial part in buffering the volumetric expansion and agglomeration of MnO_2_ nanoparticles during the discharge/charge processes. Furthermore, the large specific surface areas and mesoporous structure properties of the hollow carbon nanospheres significantly facilitate the fast transport of the lithium-ion and electrons, improving the electrochemical activities of the L-C-NSs*@*MnO_2_ electrodes. The presented work shows that the combination of specific structured lignin-based carbon nanoarchitecture with MnO_2_ provides a brand-new thought for the designation and synthesis of high-performance materials for energy-related applications.

## 1. Introduction

Lithium-ion batteries (LIBs), with large energy density, durable lifespan, and eco-friendly characteristics, are a potential energy storage system in the replacement of conventional energy devices [1,2]. The electrochemical performance of LIBs heavily relies on the electrode materials that consist of an anode and cathode. However, severe environmental problems and the rapid consumption of fossil fuel resources have prompted researchers to seek more efficient electrode materials using sustainable and renewable raw materials for LIBs. A variety of biomass-derived/based carbon matters are studied as anodic materials for LIBs; for example, cellulose-derived carbon nanofibers originating from natural cellulose substances [3], lignin-based nanofibers derived from fibrous hardwood resources [4], and nanostructured silicon/carbon composites employing the rice husk as the carbon sources and structural matrix [5], and so on.

Among the various biological substances, lignin, a natural aromatic polymer composed of benzene and methoxy, is a by-product (close to 50 million tons per year) from the papermaking and cellulosic fuels industries [6]. Lignin-based functional nanomaterials appeal to much attention on account of their excellent electrochemical activities, rich abundance of raw materials, and easy preparation [7,8,9]. Particularly, lignin-based carbon nanoarchitectures with large surface areas and pore volumes facilitate fast e^−^ transport and Li^+^ insertion during cycling. For instance, the lignin-based porous carbon nanospheres (PCN) fabricated by using the carbonization/activation approach exhibited a capacity of 300 mAh g^−1^ after 50 repeated discharge/charge cycles at a current rate of 0.1 A g^−1^ [10]. Lignin carbon fibers with disordered nano-crystalline microstructures showed a capacity of 193 mAh g^−1^ when applied as a self-supporting anode for LIBs [4]. Natural lignin-derived porous carbon modified by chloroacetic acid anode materials showed a capacity of more than 500 mAh g^−1^ at 0.05 A g^−1^, and 99% of the capacity was retained after cycled to 1000 cycles [11]. However, in spite of the improvement in electrochemical performance, the lignin-based carbon anodic materials with a relatively lower capacity and shorter cycling life are still incapable of meeting the growing needs for high energy and long lifespan electrode materials for LIBs.

Recently, to further heighten the electrochemical activities of the lignin-based carbon electrodes, methodologies like nitrogen-doped or combined with polymer or metal oxides to construct new nanoarchitectures have been proven to be effective pathways in fabricating novel lignin-based carbonaceous nanocomposites with high energy density and capacity [12,13]. It was reported that the as-prepared nitrogen-doped hard carbon, acquired through carbonization and catalysis of the lignin–melamine resins precursors, delivered a reversible capacity of 345 mAh g^−1^ at 0.1 A g^−1^ and rate capability of 145 mAh g^−1^ at 5 A g^−1^ [14]. Carbon nanofiber was produced by electrospinning of the carbon precursors consisting of lignin and polyacrylonitrile (PAN) [15] or lignin and polyethylene oxide (PEO) [16] and by way of carbonization and thermal stabilization processes. The obtained two lignin-polymer-based carbon nanofibers exhibited notably enhanced cyclic performance and high rate capability when employed as an anode for LIBs. In addition, the lignin-based carbon materials through surface modification with nanoscale metal oxides, such as MoS_2_ nanosheets [10], Fe_2_O_3_ nanoparticles [17], and NiO nanoparticles [18], displayed distinctive electrochemical activities when investigated as anodes for LIBs.

Owing to their high theoretical capacity and good safety, metal oxides, especially transition metal oxides, have been considered promising anodic materials in replacing conventional commercialized graphite anodes [19]. The conversion reaction for most of the transition metal oxides (e.g., Fe_2_O_3_, Fe_3_O_4_, MoO_3_, MnO_2_) relates to the formation of pure metal and Li_2_O during the primary insertion step and the reversible oxidation of metal after extraction [20,21,22]. Hence, because of the well-utilized oxidation state and multi-electrons involved in the conversion processes, these kinds of transition metal oxide-based electrode materials generally show high reversible capacities and discharging rates. MnO_2_, one of the transition metal oxides, has captured a great deal of attention thanks to its high storage capacity (1230 mAh g^−1^), low cost, and toxic-free properties when used as the MnO_2_-based electrodes for LIBs [23,24]. However, the low electronic conductivity and large volume expansion of the MnO_2_-based anodic matters have led to notable capacity degradation during the discharge/charge processes. One effective pathway to overcome these problems is to construct a novel nanoarchitecture consisting of the MnO_2_ and conductive carbon matrices [25,26]. It has been reported that biomass-derived carbon materials with large specific surface areas and high porosity contribute to buffering the volume expansion of the MnO_2_ nanoparticles and provide more active sites during repeated charge/discharge cycles [27,28]. For instance, a porous MnO_2_*@*carbon composite was prepared by electrodeposition of MnO_2_ on the surfaces of the corncob-derived carbon, which exhibited a relatively high capacitance of 3455 mF cm^−2^ at 1 mA cm^−2^ and a long life-shelf of 10,000 galvanostatic discharge/charge cycles when used as an asymmetric all-solid-state supercapacitor [29]. In our previous work, the MnO_2_-TiO_2_-carbon nanofiber composites were fabricated by using natural cellulose matter as a carbon source and structural scaffold. This nanocomposite with interwoven network structures and a large MnO_2_ content displayed a capacity of 677 mAh g^−1^ as cycled to 130 cycles at 0.1 A g^−1^ [30]. Therefore, the biomass-derived carbon electrodes showed superior electrochemical activities because of their porous structures and superior electrical conductivity, which facilitated the full contact of the electrode and electrolyte together with the fast transport of e^−^ and Li^+^ during the Li/Li^+^ processes. However, the problem of prolonging the cycling lifetime and realizing the highly reversible capacity of the lignin-based carbon/MnO_2_ electrode composite materials remains to be solved.

In this work, the novel lignin-based mesoporous hollow carbon nanospheres (L-C-NSs) were prepared via the extraction and carbonization processes by employing the lignosulfonate as the carbon source; the MnO_2_ nanoparticles were then uniformly loaded onto the surfaces of the as-obtained carbon nanospheres through a simple one-step mixing reaction to construct the lignin-based carbon@MnO_2_ composite. The resultant L-C-NSs*@*MnO_2_ nanosphere composites exhibited excellent long-term cycle life and highly reversible rate capability when employed as an anodic for LIBs. The significant improvement of the electrochemical performance of the L-C-NSs*@*MnO_2_ composite was attributed to the three-dimensional hollow porous structure features and large specific surface areas of the hollow lignin-based carbon nanospheres, which greatly relieved the volume expansion and pulverization of the MnO_2_ nanoparticles during the repeated cycling. What is more, the good electronic conductivity of the carbon matrix played a crucial role in shortening the pathways of Li^+^ as well as promoting the fast transport of e^−^.

## 2. Materials and Methods

### 2.1. Materials

Sodium lignosulfonate (LS) was bought from Tokyo Chemical Industry CO. Ltd. (Tokyo, Japan). Potassium permanganate (KMnO_4_, >99%) was supplied by Sigma Aldrich company (St. Louis, MO, USA). Other chemical reagents were analytical grade and used without further purification.

### 2.2. Fabrication of the Hollow L-C-NSs@MnO_2_ Nanosphere Composite

The preparation process of the hollow L-C-NSs through using lignosulfonate as the carbon source was further modified according to the reported method [18]. Firstly, 0.2 g of the sodium lignosulfonate was fully dissolved in water (10 mL) to form the homogeneous precursor solution. Then, the above mixture precursor was slowly injected into 60 mL isopropyl alcohol. After being magnetically stirred for 4 h, the as-obtained brownish-black product was centrifuged and washed with absolute ethanol. Subsequently, the L-C-NSs were acquired by carbonization of the as-obtained lignin-based nanospheres in an argon atmosphere at 800 °C for 4 h. After that, the L-C-NSs were neutralized with 0.1 M HCl to pH equal 7. To uniformly immobilize MnO_2_ nanoparticles on the surfaces of the L-C-NSs, a facile chemical redox reaction was based on the equation: 4MnO_4_^−^ + 3C + H_2_O = 4MnO_2_ + CO_3_^2−^ + 2HCO_3_^−^. Generally, 0.25 g L-C-NSs (dispersed in 100 mL water) were mixed with 0.24 g KMnO_4_ (dissolved in 30 mL de-ionized water) and underwent sonification for 0.5 h; the original black sample turned to dark brownish products. The obtained L-C-NSs*@*MnO_2_ nanocomposite was washed with H_2_O and C_2_H_5_OH several times and further dried in a vacuum oven. As a comparison, the lignin-carbon material was obtained by direct carbonization of the sodium lignosulfonate and neutralized with HCl. In addition, the MnO_2_ nanoparticles directly deposited on the surfaces of lignin-carbon to construct the lignin-carbon*@*MnO_2_ composite were prepared under the same experimental conditions.

### 2.3. Characterizations

The methods (including the specimens preparation methods for electron microscope observation) and apparatus for the tests of field emission scanning electron microscopy (FE-SEM, Hitachi SU-8010, HITACHI, Tokyo, Japan), selected area electron diffraction (SAED, JEM-2100F, JEOL, Tokyo, Japan), transmission electron microscopy (TEM, Hitachi HT-7700, HITACHI, Tokyo, Japan), Raman spectrometer (HORIBA, Paris, France), X-ray photoelectron spectra (XPS, VG Escalab Mark 2, VG Instruments, Manchester, UK), and the N_2_ adsorption–desorption analyses (Micromeritics ASAP-2020 analyzer, Micromeritics, Norcross, GA, USA) were conducted in similar ways in line with our previous report [30]. X-ray diffraction (XRD) measurements were operated on a Smartlab SE diffractometer (Rigaku Corporation, Osaka, Japan) with a Cu_Kα_ (λ = 0.15405 nm) radiation source. Thermal gravimetric analysis (TGA) was operated on Mettler Toledo STARe System TGA/DSC 3+ (Mettler Toledo Crop., Zurich, Switzerland) at a heating rate of 10 °C min^−1^ from room temperature to 800 °C in air atmosphere. Fourier transform infrared (FT-IR) spectra were tested by a Thermo Scientific Nicolet iS 20 (Thermo Fisher Scientific Crop., Waltham, MA, USA) spectrometer.

### 2.4. Electrochemical Measurements

The working electrodes were composed of active material, Super P, and polyvinylidene fluoride binder (PVDF) (mass ratio 8:1:1) and further dispersed in *N*-methyl-2-pyrrolidinone (NMP) solvent. The above mixture was uniformly coated onto nickel foam; the content of the active material was ca. 1.7–2.2 mg for each electrode. The standard CR2025 type coin cells (DodoChem, Suzhou, China) were assembled in a glove box with O_2_ and H_2_O content below 0.1 ppm; metallic Li was used as a counter electrode, and polypropylene (Celgard 2300, DodoChem, Suzhou, China) film as the separator. The electrolyte consisted of 1.0 M LiPF_6_ dissolved in ethylene carbonate (EC) and diethyl carbonate (DEC) (EC:DEC, *v*:*v* = 1:1). Cyclic voltammetry (CV) measurement was carried out on a CHI760D electrochemical workstation (scan rate: 0.1 mV s^−1^, voltage: 0.01–3.0 V, CH instruments, Shanghai, China). Galvanostatic charge/discharge performances were tested on a Neware battery testing system (Neware Technology Co., Ltd., Shenzhen, China) in a voltage range of 0.01–3.0V vs. Li/Li^+^ at a given temperature. Electrochemical impedance spectra (EIS) were tested on the CHI760D electrochemical workstation (frequency range: 100–0.01 Hz, amplitude: 5.0 mV).

## 3. Results and Discussion

### 3.1. Characterizations of the Hollow L-C-NSs@MnO_2_ Nanosphere Composite

**Figure 1:**
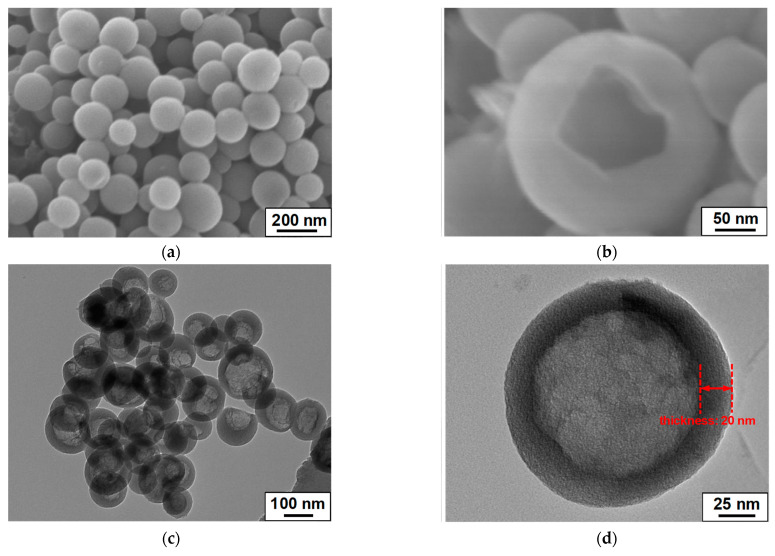
Electron micrographs of the L-C-NSs material. (**a**,**b**) FE-SEM and (**c**,**d**) TEM images of the L-C-NSs.

The L-C-NSs were fabricated by carbonization of lignin-based nanospheres by utilizing lignosulfonate as the carbon source; the MnO_2_ nanoparticles evenly grew on the surfaces of the as-prepared L-C-NSs to construct the L-C-NSs*@*MnO_2_ nanocomposite. The electron micrographs of the as-obtained L-C-NSs materials are displayed in Figure 1, and it is noticeable that the morphology structure properties of the carbon nanospheres were faithfully inherited from the lignin-based nanospheres (Figure S1, Supplementary Materials). The diameters of these L-C-NSs, which possess a smooth surface outside and a large void space inside, range from 100 to 200 nm, and the wall thickness is ca. 20 nm. The average pore size, pore volume, and the specific surface area of the carbon nanospheres are 4 nm, 0.485 cm^3^ g^−1^, and 915.2 m^2^ g^−1^, respectively (Figure S2). The mesoporous hollow structure properties of the L-C-NSs endow the material with large surface areas, which is beneficial to improving the electrochemical performances of L-C-NSs-based electrodes. The lignin-carbon*@*MnO_2_ composite, on the other hand, was prepared by immobilizing MnO_2_ nanoparticles on the surfaces of lignin-based carbon obtained through direct carbonization of the lignosulfonate. However, the original lignin-based carbon inclines to agglomerate into bulk forms with lots of mesopores and macropores on the surfaces (Figure S3). In addition, the porous structure properties of the lignin-carbon material were clearly seen due to the decomposition of small molecules as well as the evaporation of H_2_O during the carbonization process [31], and the specific surface area and pore volume are as low as 51.5 m^2^ g^−1^ and 0.205 cm^3^ g^−1^ (Figure S2). It was believed that the L-C-NSs with higher surface areas and larger pore volume facilitate the improvement of the cycling capacity and the rate capability of the L-C-NSs-based composite when used as anodic for LIBs.

**Figure 2:**
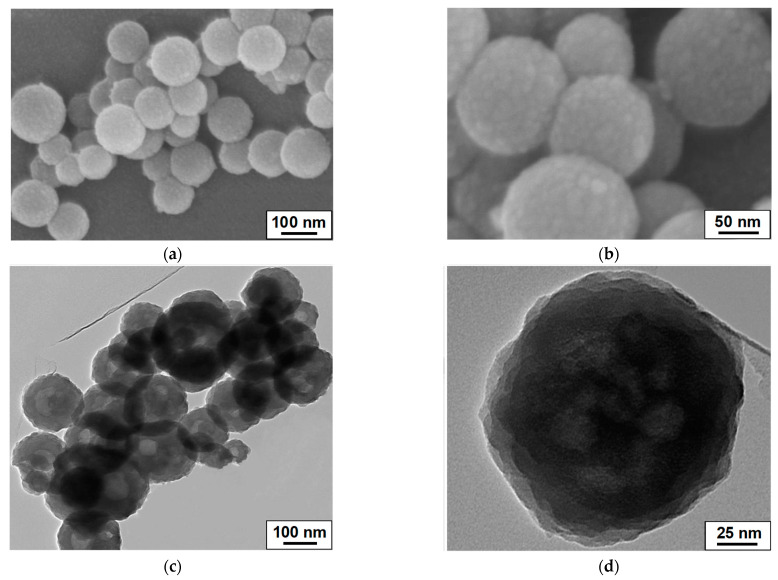
Electron micrographs of the L-C-NSs@MnO_2_ nanocomposite. (**a**,**b**) SEM and (**c**,**d**) TEM images of the L-C-NSs@MnO_2_ composite at different magnifications.

**Figure 3:**
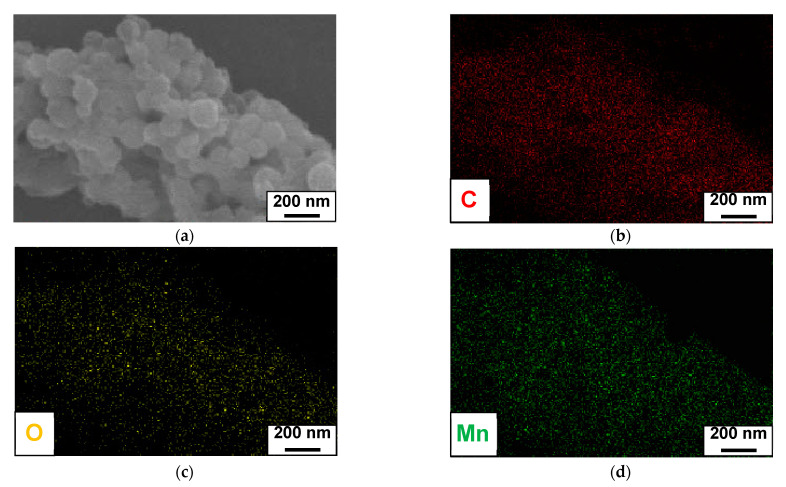
SEM images of an individual L-C-NSs@MnO_2_ composite (**a**) with the corresponding EDS elemental mapping of (**b**) C, (**c**) O, and (**d**) Mn.

The immobilization of the MnO_2_ nanoparticles on the surfaces of L-C-NSs resulted in the L-C-NSs@MnO_2_ composite, and the original spherical morphologies of L-C-NSs were maintained by the nanocomposites. The MnO_2_ nanoparticles with a diameter of 10~20 nm uniformly grew on the surfaces of the L-C-NSs (Figure 2a,b). The diameter of an individual L-C-NSs*@*MnO_2_ nanosphere composite was in the range of 100~200 nm, and its rough surfaces and mesoporous hollow structures were observed (Figure 2c,d). The elemental mapping of the elements C, O, and Mn in the L-C-NSs@MnO_2_ is displayed in Figure 3, which further verifies the successful immobilization of MnO_2_ nanoparticles on the surfaces of the nanocomposite. The three-dimensional porous hollow structures of the L-C-NSs@MnO_2_ provide more active sites and facilitate the transfer of Li^+^ and e^−^ during the lithiation/delithiation processes. In comparison, the bulk lignin-carbon material was covered by a thick MnO_2_ layer, and the MnO_2_ nanoparticles tend to agglomerate together to form larger particles (Figure S3).

**Figure 4:**
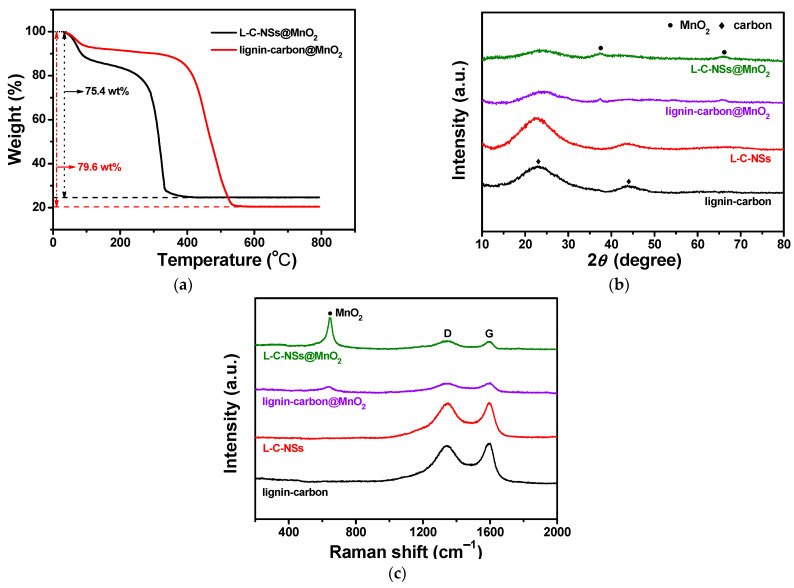
(**a**) TGA curves of the L-C-NSs@MnO_2_ and lignin-carbon@MnO_2_ composites. (**b**) XRD patterns and (**c**) Raman spectra of the corresponding L-C-NSs@MnO_2_, lignin-carbon@MnO_2_, L-C-NSs, and lignin-carbon materials.

As verified by the TG test, the mass contents of Mn element in L-C-NSs*@*MnO_2_ and lignin-carbon@MnO_2_ nanocomposite are 24.6 wt% and 20.4 wt%, respectively (Figure 4a). The EDS measurements of the elements C, O, and Mn in the L-C-NSs*@*MnO_2_ composite are 54.419, 21.064, and 24.517 wt%, respectively. These results are in agreement with the TG tests (Figure S4a). Figure 4b exhibited the XRD patterns of the lignin-based carbon materials. The two weak peaks of the L-C-NSs*@*MnO_2_ and lignin-carbon*@*MnO_2_ composites appeared at 37.3° and 65.6° and were indexed to the (−111) and (020) planes of the *α*-MnO_2_, respectively (JCPDS#. 80–1098) [30,32]. Regarding the L-C-NSs and lignin-carbon materials, two broad diffraction peaks are indexed to graphitic carbon located at 24° (002 plane) and 44° (100 plane) [33]. The peak intensity of the carbon phase in the two lignin-based MnO_2_ composites was weaker than those of the lignin-carbon and L-C-NSs. Interestingly, it was very hard to find the graphitic carbon in the two composites, which was ascribed to the consumption of the carbon during the generation of MnO_2_ nanoparticles [30]. The structure properties of the corresponding samples were further confirmed by Raman spectra, as revealed in Figure 4c. A broad peak that presented at 650 cm^−1^ in the L-C-NSs@MnO_2_ and lignin-carbon@MnO_2_ composites was indexed to the stretching vibration of Mn-O of the MnO_6_ octahedra [34]. All samples showed disordered amorphous (D-band, 1350 cm^−1^) and graphitic (G-band, 1580 cm^−1^) carbon bands, which were ascribed to the disorder and graphitic band of carbon, respectively [35]. It was noticed that the peak intensity of MnO_2_ in the L-C-NSs@MnO_2_ was stronger than that of the lignin-carbon@MnO_2_ composite, indicating higher MnO_2_ content in the L-C-NSs@MnO_2_ composite. FT-IR was used to further investigate the functional groups of the lignin-based carbon (before carbonization) and L-C-NSs (after carbonization). In the FT-IR spectrum (Figure S4b), the absorption band at 3448 cm^−1^ is caused by the stretching vibration of hydroxyl, while the peaks at 2938 and 1604 cm^−1^ are attributed to the stretching vibrations of the C-H and C=O bonds, respectively. The peaks located at 1511 and 1459 cm^−1^ refer to the vibration of benzene. The peaks near 621 cm^−1^ are the stretching vibration of O-S-O. The peaks at 3448, 2364, and 1685 cm^−1^ of the L-C-NSs are indexed to the stretching vibration of C=O and O=C=O, C=C, respectively, while the peaks at 1458 and 879 cm^−1^ are ascribed to the bending vibrations of the C-H and S=O bonds, respectively.

**Figure 5:**
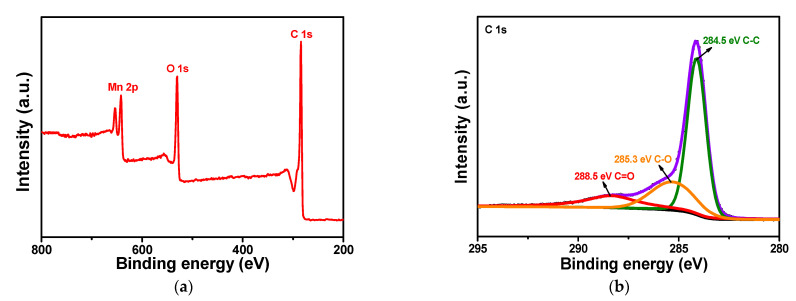

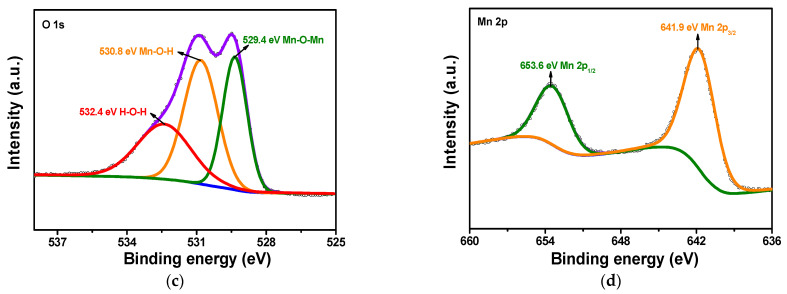
(**a**) The XPS survey spectrum of the L-C-NSs@MnO_2_ composite and high-resolution XPS spectra of (**b**) C 1s, (**c**) O 1s, and (**d**) Mn 2p.

The chemical state of elements C, O, and Mn in the L-C-NSs@MnO_2_ was evaluated by XPS spectra. As shown in Figure 5a, the Mn 2p peak was observed along with the O 1s and Mn 2p, indicating the presence of carbon, oxygen, and manganese in the composite. A high-resolution XPS spectrum of C 1s was exhibited in Figure 5b; the peaks presented at 284.5, 285.3, and 288.5 eV were assigned to the C–C, C–O, and C=O, respectively [29,36]. Figure 5c exhibited the O 1s spectrum, where one peak centered at 529.4 eV and two relatively broad peaks appeared at 530.8 and 532.4 eV were attributed to the Mn–O–Mn, Mn–O–H and H–O–H, respectively [29]. The results were in accordance with reported data (529.3~530.3 eV: oxide, 530.5~531.5 eV: hydroxide, 531.8~532.8 eV: water) [37]. The two main peaks appeared at 641.9 and 653.6 eV, corresponding to Mn 2p_3/2_ and Mn 2p_1/2_, respectively (Figure 5d). The differential peak value of 11.7 eV between the two peaks was in good agreement with the MnO_2_, demonstrating the +4 valence state of Mn [38]. Moreover, the whole XPS spectrum together with C(1s), O(1s), and S(2p) regions of the L-C-NSs are shown in Figure S5, and the peak of S 2p originated from the lignosulfonate during the carbonization process, proving the existence of elements C, O, S in the as-obtained carbon nanospheres [18]. Moreover, the EDS tests of element S in the L-C-NSs were measured to be 14.3 wt%. However, after the L-C-NSs were immobilized with MnO_2_ nanoparticles, the element S contained in the as-resultant L-C-NSs@MnO_2_ nanocomposite decreased to 0.5 wt%, which was hardly detected by the XPS characterization.

### 3.2. Electrochemical Properties of the Porous Hollow L-C-NSs@MnO_2_ Nanosphere Composite

**Figure 6:**
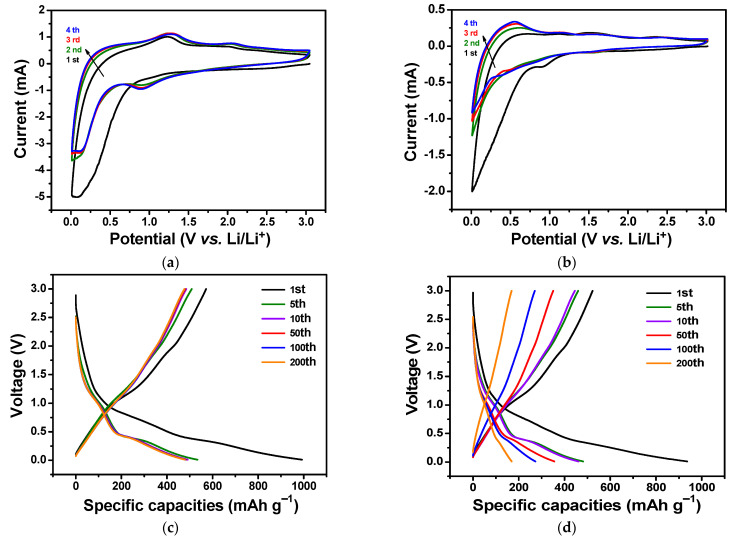
CV curves of the initial four cycles of (**a**) L-C-NSs*@*MnO_2_ and (**b**) lignin-carbon*@*MnO_2_ electrodes tested at a scan rate of 0.5 mV s^−1^ between 0.01 and 3 V. Galvanostatic charge/discharge profiles of the (**c**) L-C-NSs*@*MnO_2_ and (**d**) lignin-carbon*@*MnO_2_ composites at a current density of 0.1 A g^−1^ for different cycles.

The well-defined porous structures of the L-C-NSs and the high content of the MnO_2_ impart the L-C-NSs*@*MnO_2_ composite with excellent electrochemical activities when employed as anodes for LIBs. Figure 6a shows the CV curves of the L-C-NSs*@*MnO_2_ composite for the first four discharge/charge cycles. In the initial cathodic curve, a broad peak at lower voltage and a mild peak near 0.8 V were ascribed to the generation of a solid electrolyte interface (SEI) layer and the reduction of MnO_2_ with Li ions [30,39]. Two anodic peaks were observed at 1.24 and 2.02 V in the first charge cycle and were attributed to the two-step oxidation reaction of Mn^0^ to Mn^2+^ and Mn^2+^ to Mn^4+^ and the decomposition of electrolyte [40]. In the next three Li^+^ insertion/extraction processes, the original voltage at 0.1 V disappeared, the reduction peaks shifted to ca. 0.9 V, and the oxidation peaks shifted to 2.05 V, demonstrating the irreversible capacity loss on account of the formation of SEI layer and Li_2_O. Interestingly, the redox peaks, as well as the peak intensities of CV curves, exhibited good repeatability in subsequent cycles, indicating excellent cycling capacity and structural stability of the L-C-NSs*@*MnO_2_ nanocomposite. In contrast, the lignin-carbon*@*MnO_2_ composite in Figure 6b, where in the first cycle, a weak peak at 0.3 V and a broad peak located at 0.9 V were attributed to the formation of SEI film and the reduction of Mn^2+^ to Mn^0^, respectively. Two mild oxidation peaks at 1.50 and 2.38 V were observed in the primary charge curves, suggesting the electrochemical reaction of Mn^0^ to Mn^4+^ occurred in two steps [32]. In the subsequent discharge/charge cycles, the lower voltage peak was missed due to the formation of the SEI layer that occurred in the first cycle. The redox peaks shifted to a higher voltage, and the intensities decreased rapidly, implying the irreversible capacity fading and the structural collapse of the electrode.

The galvanostatic charge/discharge profiles of the L-C-NSs*@*MnO_2_ composite are shown in Figure 6c, which delivers the first discharge and charge capacities of 993 and 587 mAh g^−1^, respectively, corresponding to a Coulombic efficiency of 59.1%. In contrast, the lignin-carbon*@*MnO_2_ composite exhibited an initial discharge and charge capacity of 936 and 520 mAh g^−1^, respectively, showing a Coulombic efficiency of 55.6% (Figure 6d). The irreversible capacity loss of the lignin-carbon*@*MnO_2_ was larger than that of the L-C-NSs*@*MnO_2_ anode due to the formation of the SEI film as well as the less content of MnO_2_ contained in the former composite [30]. The discharge capacity of the L-C-NSs*@*MnO_2_ decreased to 534 and 484 mAh g^−1^ when cycled to the 5th and 50th cycle, respectively, and stabilized at 479 mAh g^−1^ by the 200th cycle. A flat voltage plateau at around 0.3–0.5 V and a sharp plateau at about 0.9–1.2 V in the discharge curves corresponded to the electrochemical reaction of the MnO_2_ with Li. Two mild charge plateaus at 1.0–1.3 and 1.8–2.1 V were attributed to the two-step oxidation of Mn^0^ to Mn^4+^ [41]. However, the capacity of the lignin-carbon*@*MnO_2_ anode material decreased sharply from 482 to 356 mAh g^−1^ at the 5th and 50th cycle, respectively, and only reached 169 mAh g^−1^ after 200 discharge/charge cycles. In comparison, the capacities of the L-C-NSs dropped quickly from 681 to 230 mAh g^−1^ in the initial five cycles and remained at 220 mAh g^−1^ afterward (Figure S6). Nevertheless, the capacities of the lignin-carbon materials were reduced to only 97 mAh g^−1^ as cycled to the 200th cycle (Figure S6). It was noticed that the L-C-NSs*@*MnO_2_ electrode showed higher capacity and capacity retention capability than those of the other three counterpart anode materials, which was ascribed to the structural stability of the original three-dimensional L-C-NSs support and the high content of MnO_2_ in the composite, thus, the L-C-NSs*@*MnO_2_ nanocomposites displayed better electrochemical activities.

**Figure 7:**
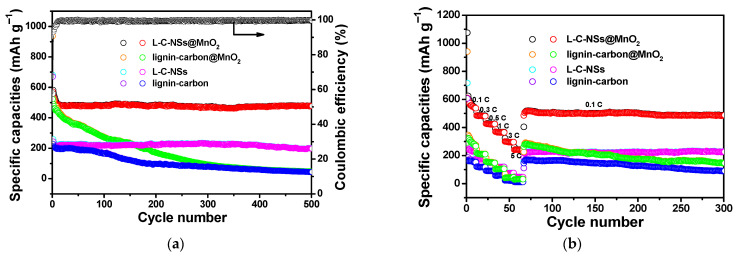
(**a**) Long-life cycling performances of the L-C-NSs*@*MnO_2_, lignin-carbon*@*MnO_2_, L-C-NSs, and lignin-carbon electrode materials at a current rate of 0.1 A g^−1^ as well as the Coulombic efficiency curve of the L-C-NSs*@*MnO_2_ nanocomposite. (**b**) The rate capability of the L-C-NSs*@*MnO_2_, lignin-carbon*@*MnO_2_, L-C-NSs, and lignin-carbon at varied current densities from 0.1 to 5.0 C.

The cycling performances of the L-C-NSs*@*MnO_2_, lignin-carbon*@*MnO_2_, L-C-NSs, and lignin-carbon electrode materials at a current rate of 0.1 A g^−1^ are displayed in Figure 7a. The capacity of the L-C-NSs*@*MnO_2_ nanocomposite decreased rapidly in the initial 5 cycles and maintained at 500 mAh g^−1^ by the 8th cycle; a capacity of 478 mAh g^−1^ was obtained after 500 long-term discharge/charge cycles, which was equivalent to a capacity degradation rate of 0.036% per cycle thereafter. As a comparison, the capacity of the lignin-carbon*@*MnO_2_ composite dropped suddenly from 936 to 92 mAh g^−1^ after 300 repeated Li^+^ insertion/extraction cycles and finally decreased to less than 50 mAh g^−1^ by the 500th cycle. The capacity of the L-C-NSs electrode quickly stabilized at 220 mAh g^−1^ after the initial 5 cycles and even remained unchanged as cycled to 500 cycles. The initial discharge and charge capacities of the lignin-carbon material were 670 and 228 mAh g^−1^, respectively, showing a Coulombic efficiency of 34%, and its capacity reduced to less than 50 mAh g^−1^ after 500 cycles. It was found that the L-C-NSs*@*MnO_2_ composite showed higher capacity and better cycling stability than the other three materials, and the capacity contribution of the MnO_2_ in the L-C-NSs*@*MnO_2_ composite was estimated to be 1268.8 mAh g^−1^, corresponding to 103.2% of the theoretical capacity of MnO_2_. Compared to the other reported lignin-based carbon nanomaterials and MnO_2_-based carbon composite, the current fabricated hollow L-C-NSs*@*MnO_2_ nanospheres exhibited either a longer cycling lifetime or a higher capacity at comparable current densities (Table 1). These improvements are attributed to the hollow three-dimensional porous structures and the large surface areas of the L-C-NSs matrix, which effectively facilitate electron transport and shorten the diffusion pathway of Li^+^ during the discharge/charge processes. Moreover, the volume expansion and pulverization of the immobilized MnO_2_ nanoparticles of the L-C-NSs*@*MnO_2_ composite are greatly alleviated by the unique hollow structure of L-C-NSs, therefore, the composite with higher MnO_2_ content displayed significantly enhanced capacity retention capability and excellent long-term cycling lifespan.

To further evaluate the electrochemical advantages of the L-C-NSs*@*MnO_2_ material, the rate capabilities of the nanocomposite at different current rates are exhibited in Figure 7b. The discharge capacities of the composite were kept at 558, 485, 425, 367, 296, 243 mAh g^−1^ as the current rate was 0.1, 0.3, 0.5, 1, 3, 5 C (1 C = 1 A g^−1^), respectively. When the current rate was reset to 0.1 C, a reversible capacity of 505 mAh g^−1^ was acquired and kept slightly changing until 300 discharge/charge cycles. The capacity degradation rate was ca. 0.026% per cycle after long-term and high-rate Li^+^ insertion/extraction processes. However, the lignin-carbon*@*MnO_2_ electrode material only delivered a capacity of 282, 210, 151, 104, 43, and 31 mAh g^−1^ at the current density of 0.1, 0.3, 0.5, 1, 3, and 5 C, respectively. Although the capacities of the L-C-NSs materials were comparable to or less than that of the lignin-carbon*@*MnO_2_ in lower current rates, the L-C-NSs showed much higher capacities as the current rate in the range of 1 C–5 C as well as being set back to 0.1 C. These results demonstrated that the L-C-NSs with high porosity and hollow three-dimensional porous structures are beneficial for the L-C-NSs*@*MnO_2_ to maintain the structural integrity in the large current rate discharge/charge processes. As for the lignin-carbon anode, it showed the lowest capacities at the same current densities, and the capacity was close to the X axial at a higher current rate. Apparently, the L-C-NSs*@*MnO_2_ composite showed higher cycling capacity and superior reversible capacity when compared to the other three electrode materials. The results further verified that the distinct hollow nanospheres with mesoporous structures and high surface-to-volume ratio facilitated the improvement of the rate performance of the composite during varied current densities.

**Figure 8:**
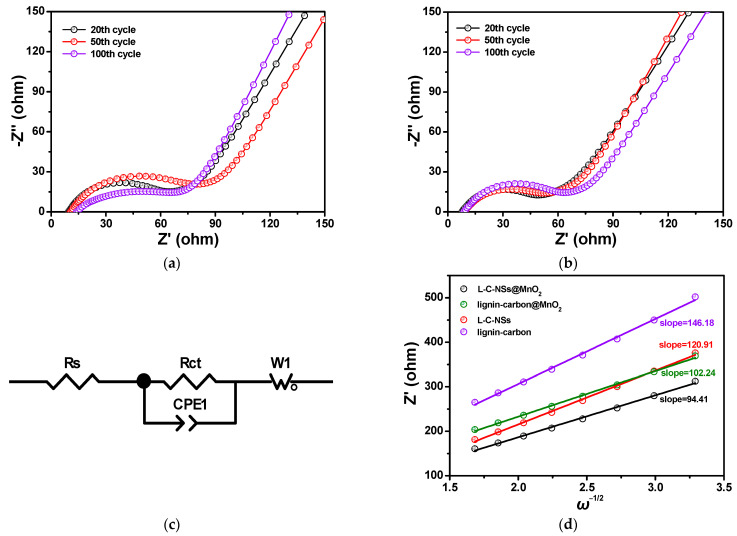
The Nyquist plots of (**a**) the L-C-NSs*@*MnO_2_ and (**b**) lignin-carbon*@*MnO_2_ electrodes after charging to 3.0 V at the 20th, 50th, and 100th cycle. (**c**) The equivalent circuit model utilized to fit the impedance spectra. (**d**) Relationship between Z′ and *ω*^−1/2^ plots at the low frequency.

To better explore the electrochemical reaction kinetics and study the significantly improved electrochemical activities of the L-C-NSs*@*MnO_2_ composite, Figure 8 shows the Nyquist plots of the L-C-NSs*@*MnO_2_ and lignin-carbon*@*MnO_2_ electrodes after 10, 50, and 100 repeated discharge/charge cycles obtained from the electrochemical impedance spectroscope (EIS) tests. It was demonstrated that L-C-NSs*@*MnO_2_ material displayed a smaller diameter of the semicircle in the high-frequency region and a sharper slope line in the low-frequency region at paralleled cycles when compared to the lignin-carbon*@*MnO_2_ composite, indicating a lower resistance and better reaction kinetic process of the electrode [45,46]. The kinetic parameters of two electrodes at different cycles were calculated utilizing the equivalent circuit, and the charge transfer impedance (*R_ct_*) values of the L-C-NSs*@*MnO_2_ were calculated to be 29.6 and 40.5 Ω at the 10th and 100th cycle, respectively, whereas the *R_ct_* value of lignin-carbon*@*MnO_2_ composite increased from 41.3 to 65.2 Ω (Table 2). In spite of the slight differences in the *R_s_* values of the four samples, the *R_ct_* value of the L-C-NSs*@*MnO_2_, lignin-carbon*@*MnO_2_, L-C-NSs, and lignin-carbon electrodes at the 50th discharge/charge cycle were estimated to be 32.3, 50.9, 88.6, 107.4 Ω, respectively (Table 2, Figure S7, Table S1). Obviously, the L-C-NSs*@*MnO_2_ composite showed the lowest *R_ct_* values, which further verified that the electrode had a high-speed charge transfer and fast Li^+^ diffusion processes, resulting in a long battery cycling lifetime and highly reversible rate performances of the electrode. In addition, the straight line in the low-frequency region of each anode implied that the impedance was related to the lithium-ion diffusion coefficient ($D_{Li}{}_{{}^{+}}$), and the $D_{Li}{}_{{}^{+}}$ was calculated by employing the following two equations [36,47]:

Z′ = R_e_ + R_C_ + *σ_w_ω*^1/2^ (1)

(2) $$D_{\mathrm{Li}}{}_{{}^{+}}=R^{2}T^{2}/2n^{4}A^{2}F^{4}C^{2}\sigma_{w}^{2}$$

where *R* represents the gas constant, *T* refers to the absolute temperature, *A* represents the contact area of the electrode, *n* indicates the transferred number of electrons, *F* represents the Faraday constant, *C* is the concentration of Li^+^, *ω* is the angular frequency, and *σ_w_* refers to the Warburg factor. According to Equation (1), the *σ_w_* is acquired from the slope of the line and exhibited in Figure 8d, and the values were measured to be 94.41, 102.24, 120.91, and 146.18 Ω cm^2^ s^−1/2^ for the L-C-NSs*@*MnO_2_, lignin-carbon*@*MnO_2_, L-C-NSs, and lignin-carbon electrodes, respectively. Therefore, the Li^+^ diffusion coefficient ($D_{Li}{}_{{}^{+}}$) was calculated on the basis of Equation (2), and the values of the $D_{Li}{}_{{}^{+}}$ were 2.04 × 10^−15^, 1.74 × 10^−15^, 1.25 × 10^−15^, and 8.53 × 10^−16^ cm^2^ s^−1^ for the four electrodes mentioned above, respectively. The results demonstrated that the L-C-NSs*@*MnO_2_ composite with exquisite porous structures can greatly enhance the efficiency of Li^+^ transport and improve the electronic conductivity of the electrode. The SEM image of the L-C-NSs*@*MnO_2_ electrode after 300 repeated Li^+^ insertion/extraction processes is exhibited in Figure S8. The original spherical structure of the nanocomposite was maintained after longtime cycling, and the MnO_2_ nanoparticles were tightly immobilized on the surfaces of the L-C-NSs, proving the structural integrity contributed to the excellent chemical performances of the L-C-NSs*@*MnO_2_.

## 4. Conclusions

The lignin-based L-C-NSs*@*MnO_2_ nanocomposite was fabricated by immobilizing the MnO_2_ nanoparticles on the surfaces of the hollow L-C-NSs nanospheres by using the lignosulfonate as the carbon source. The three-dimensional hollow porous structure properties of L-C-NSs material were faithfully maintained by the L-C-NSs*@*MnO_2_ composite. When utilized as an anodic material for LIBs, the composite exhibited excellent cycling capacity and prolonged lifespan, as well as highly reversible rate capability. The remarkably improved electrochemical performances of the L-C-NSs*@*MnO_2_ composite are ascribed to the synergistic effect of the well-defined L-C-NSs structural matrix and the high content of immobilized MnO_2_ nanoparticles, of which the hollow L-C-NSs promoted the kinetic process of electrochemical reaction, in addition, the MnO_2_ with high theoretical capacity contributed to the increase in electrode capacity. The present work holds great potential for the development of functional nanostructured composite prepared by employing renewable biosources as carbon sources in energy-related fields.

## Figures and Tables

**Table 1:** The comparison of electrochemical performances of the present hollow L-C-NSs@MnO_2_ nanosphere composite with other reported lignin-based carbon and MnO_2_-based carbon composite anode materials for LIBs.

Materials	Current Density (mA g^−1^)	Cycle Number	Specific Capacity (mAh g^−1^)	Ref.
Lignin carbon fibers	15	70	193	[4]
Lignin-based porous carbon nanospheres	100	50	300	[10]
MWNTS@MnO_2_	100	100	~200	[42]
MnO_2_ nanorods/3D-rGO	100	60	595	[43]
Coaxial MnO_2_/carbon nanotube	50	15	500	[44]
MnO_2_-rGO-CNTs	100	200	380.9	[45]
Hollow L-C-NSs@MnO_2_ nanospheres	100	500	478	This work

**Table 2:** Equivalent circuit parameters acquired from fitting the experimental impedance spectra of the L-C-NSs*@*MnO_2_ and lignin-carbon*@*MnO_2_ composite electrodes.

Samples	Cycle	*R_s_* (Ω)	*R_ct_* (Ω)
L-C-NSs@MnO_2_	20th	7.3	29.6
	50th	8.6	32.3
	100th	8.7	40.5
	20th	8.8	41.3
lignin-carbon@MnO_2_	50th	9.6	50.9
	100th	11.9	65.2

## Data Availability

The data that support the findings of this study are available on request from the corresponding author.

## Supplementary Materials

The following are available online at https://www.mdpi.com/article/10.3390/ma16237283/s1, Figure S1: SEM images of the lignin-based nanospheres at different magnifications. Figure S2: (a) N_2_ adsorption-desorption isotherm and (b) pore-size distribution curve of the L-C-NSs; (c) N_2_ adsorption-desorption isotherm and (d) pore-size distribution curve of the lignin-carbon. Figure S3: (a) SEM and (b) TEM images of the lignin-carbon material; (c) SEM and (d) TEM images of the lignin-carbon@MnO_2_ composite. Figure S4: Energy dispersive X-ray (EDX) microanalysis report of the L-C-NSs*@*MnO_2_ composites. Figure S5: The XPS survey spectrum of (a) the L-C-NSs material and high-resolution XPS spectra of (b) C 1s, (c) O 1s, and (d) S 2p. Figure S6: Galvanostatic charge/discharge profiles of the (a) L-C-NSs and (b) lignin-carbon at a current rate of 0.1 A g^−1^ for different cycles. Figure S7: The Nyquist plots of the L-C-NSs and lignin-carbon electrodes after charging to 3.0 V at the 50th cycle. Table S1: Equivalent circuit parameters obtained from fitting the experimental impedance spectra of the L-C-NSs and lignin-carbon electrodes. Figure S8: SEM image of the L-C-NSs*@*MnO_2_ electrode after 300 repeated discharge/charge cycles.
