# Supplementary material for: Lignin-Based Mesoporous Hollow Carbon@MnO2 Nanosphere Composite as an Anodic Material for Lithium-Ion Batteries
Source: Materials (Basel). 2023 Nov 23;16(23):7283. doi: 10.3390/ma16237283 (PMC10707017; doi:10.3390/ma16237283)
Supplement: Supplementary file 1 [file materials-16-07283-s001.zip › materials-2723065-supplementary.pdf]

Supplementary Materials

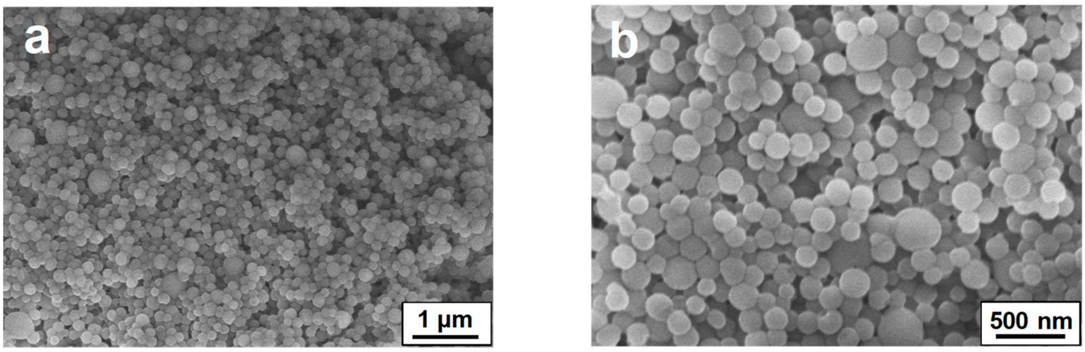

Figure S1. SEM images of the lignin-based nanospheres at different magnifications.

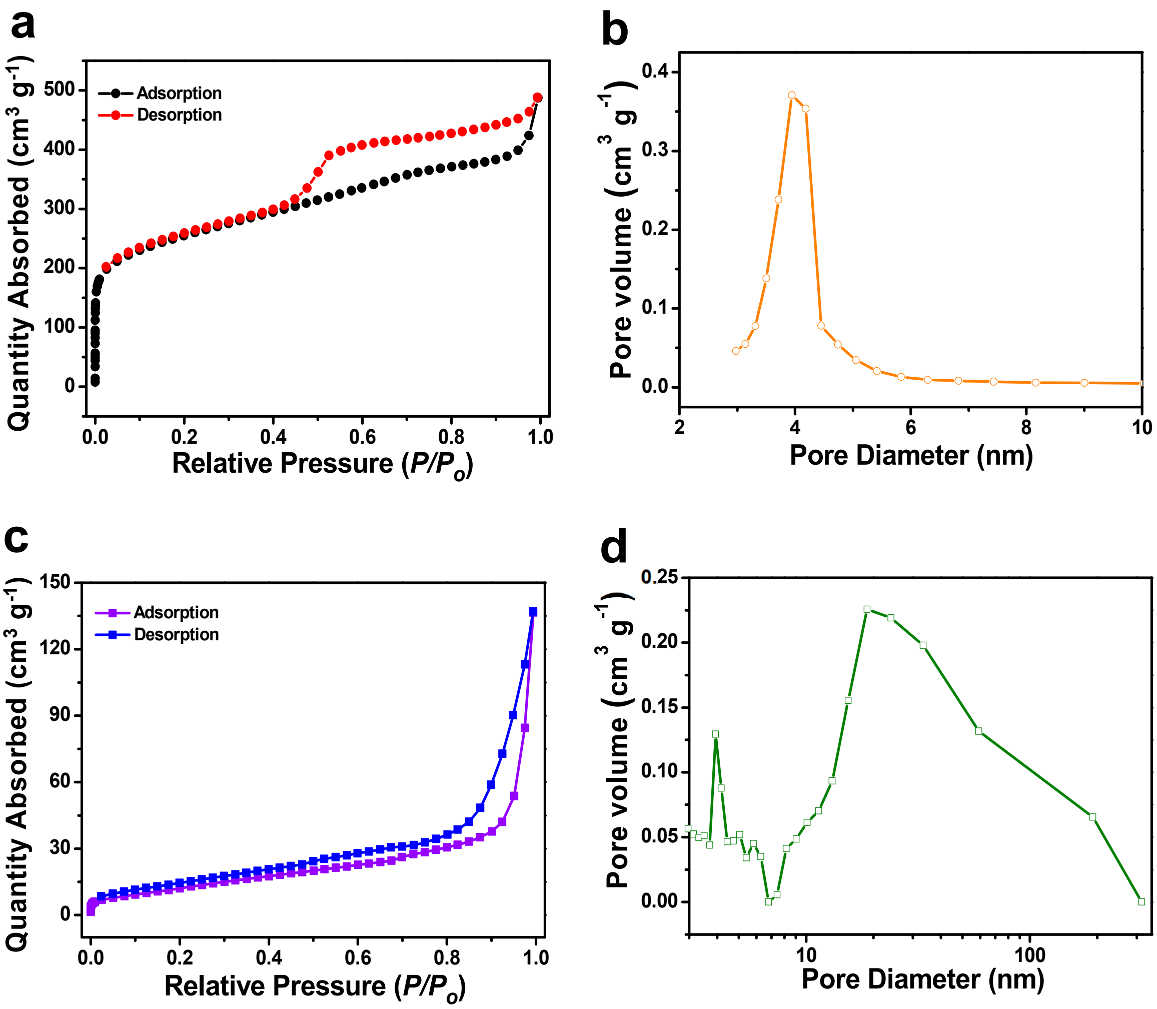

Figure S2. (a) N_2 adsorption-desorption isotherm and (b) pore-size distribution curve of the L-C-NSs; (c) N_2 adsorption-desorption isotherm and (d) pore-size distribution curve of the lignin-carbon.

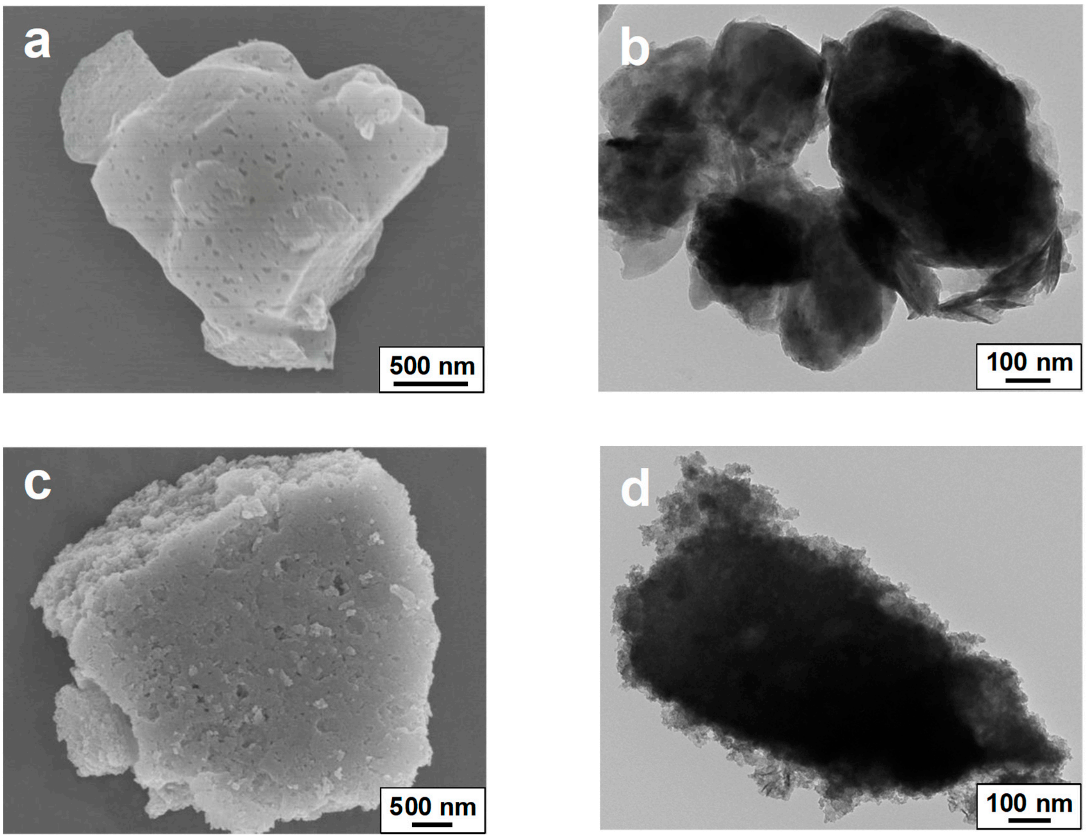

Figure S3. (a) SEM and (b) TEM images of the lignin-carbon material; (c) SEM and (d) TEM images of the lignin-carbon@MnO_2 composite.

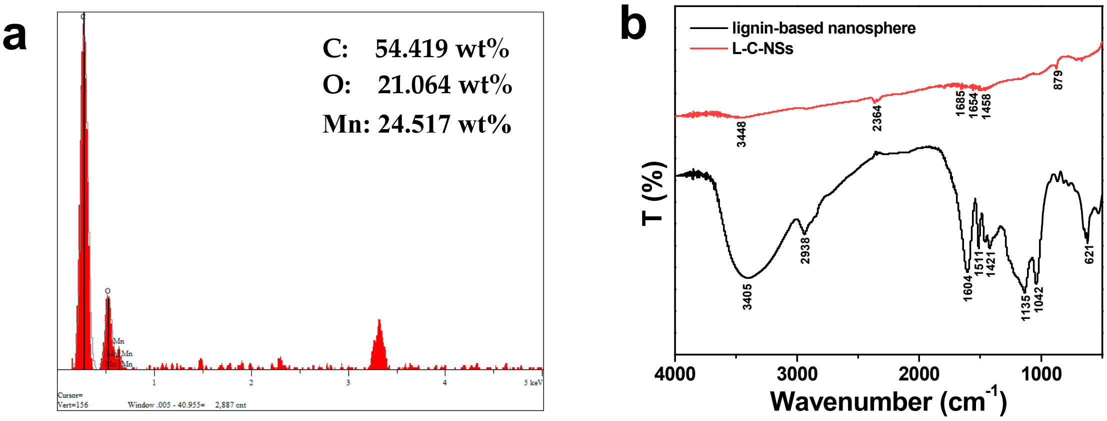

Figure S4. (a) Energy dispersive X-ray (EDX) microanalysis report of the L-C-NSs@MnO_2 composites. (b) FT-IR spectra of the lignin-based nanosphere and L-C-NSs material.

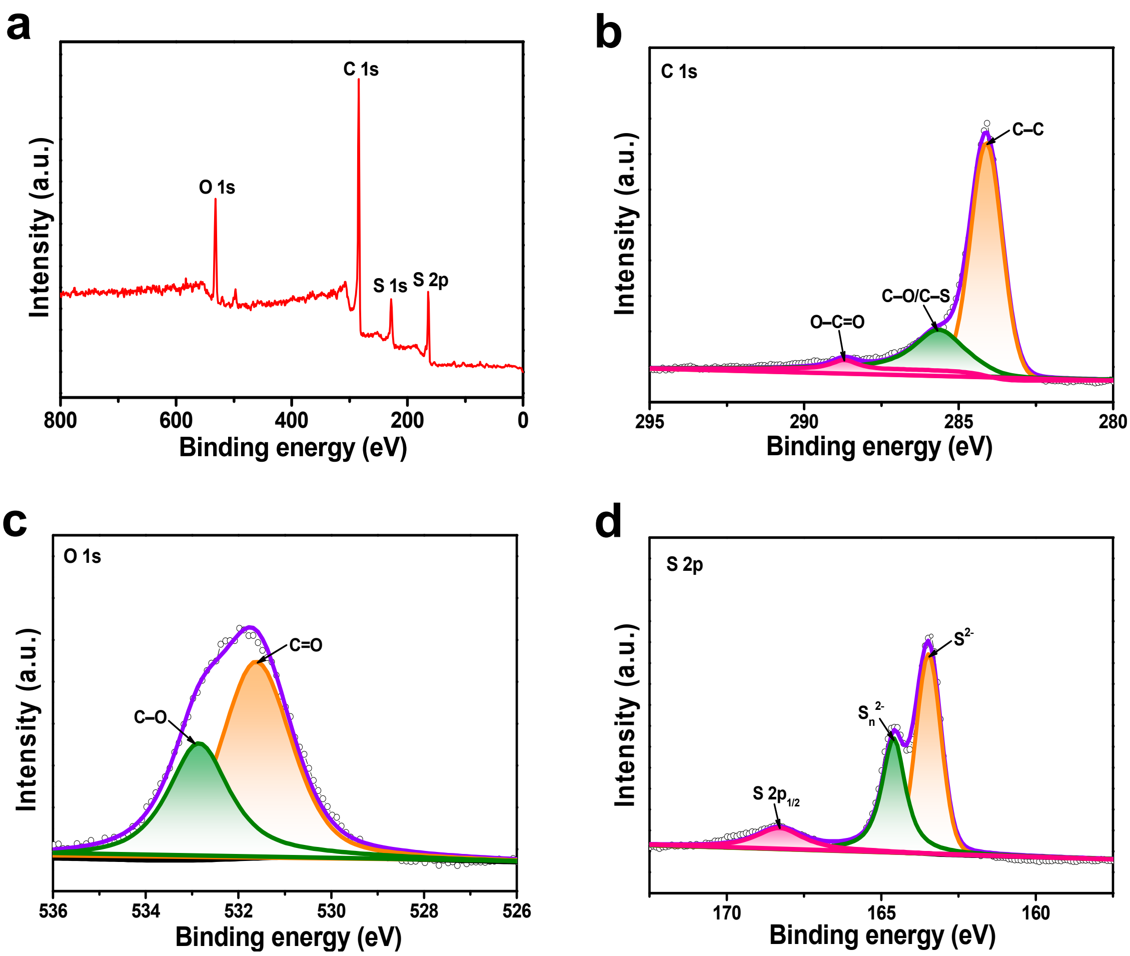

Figure S5. The XPS survey spectrum of (a) the L-C-NSs material, and high-resolution XPS spectra of (b) C 1s, (c) O 1s, and (d) S 2p.

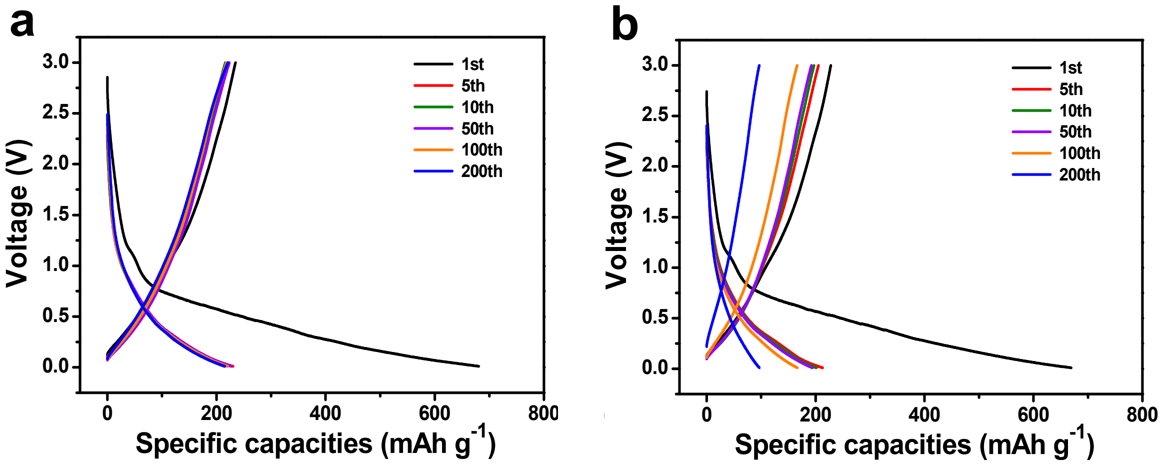

Figure S6. Galvanostatic charge/discharge profiles of the (a) L-C-NSs and (b) lignin-carbon at a current rate of 0.1 A g^{-1} for different cycles.

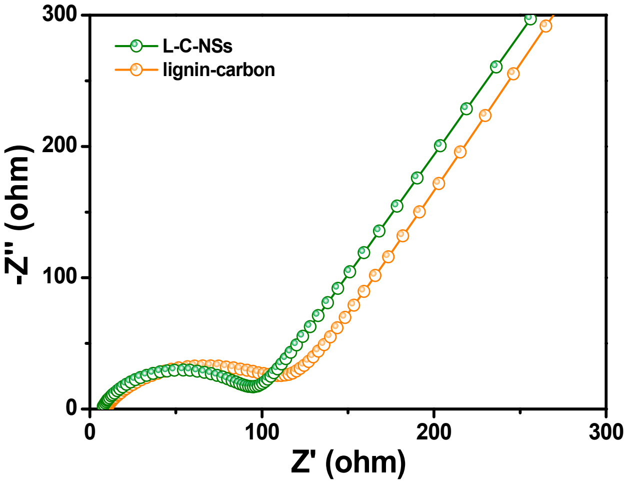

Figure S7. The Nyquist plots of the L-C-NSs and lignin-carbon electrodes after charging to 3.0 V at the 50th cycle.

Table S1. Equivalent circuit parameters obtained from fitting the experimental impedance spectra of the L-C-NSs and lignin-carbon electrodes.

**samples**	R_s (Ω)	R_{ct} (Ω)
L-C-NSs	6.586	88.6
lignin-carbon	8.662	107.4

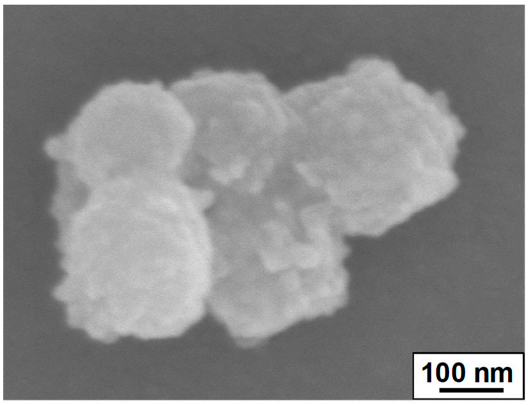

Figure S8. SEM image of the L-C-NSs@MnO_2 electrode after 300 repeated discharge/charge cycles.
